# Utilization and Acceptability of Formal and Informal Support for Adolescents Following Self-Harm Before and During the First COVID-19 Lockdown: Results From a Large-Scale English Schools Survey

**DOI:** 10.3389/fpsyt.2022.881248

**Published:** 2022-06-24

**Authors:** Galit Geulayov, Rohan Borschmann, Karen L. Mansfield, Keith Hawton, Paul Moran, Mina Fazel

**Affiliations:** ^1^Centre for Suicide Research, Department of Psychiatry, University of Oxford, Warneford Hospital, Oxford, United Kingdom; ^2^Justice Health Unit (Centre for Health Equity), Melbourne School of Population and Global Health, University of Melbourne, Melbourne, VIC, Australia; ^3^Centre for Adolescent Health, Murdoch Children's Research Institute, Melbourne, VIC, Australia; ^4^Melbourne School of Psychological Sciences, The University of Melbourne, Melbourne, VIC, Australia; ^5^Department of Psychiatry, University of Oxford, Warneford Hospital, Oxford, United Kingdom; ^6^Oxford Health NHS Foundation Trust, Warneford Hospital, Oxford, United Kingdom; ^7^Centre for Academic Mental Health, Department of Population Health Sciences, Bristol Medical School, University of Bristol, Bristol, United Kingdom

**Keywords:** Self-harm, adolescence, school, help-seeking, mental health, self-poisoning, self-injury

## Abstract

**Background:**

Little is known about the perceived acceptability and usefulness of supports that adolescents have accessed following self-harm, especially since the onset of the COVID-19 pandemic.

**Aims:**

To examine the utilization and acceptability of formal, informal, and online support accessed by adolescents following self-harm before and during the pandemic.

**Method:**

Cross-sectional survey (OxWell) of 10,560 secondary school students aged 12–18 years in the south of England. Information on self-harm, support(s) accessed after self-harm, and satisfaction with support received were obtained *via* a structured, self-report questionnaire. No tests for significance were conducted.

**Results:**

1,457 (12.5%) students reported having ever self-harmed and 789 (6.7%) reported self-harming during the first national lockdown. Informal sources of support were accessed by the greatest proportion of respondents (friends: 35.9%; parents: 25.0%). Formal sources of support were accessed by considerably fewer respondents (Child and Adolescent Mental Health Services: 12.1%; psychologist/ psychiatrist: 10.2%; general practitioner: 7.4%). Online support was accessed by 8.6% of respondents, and 38.3% reported accessing no support at all. Informal sources of support were rated as most helpful, followed by formal sources, and online support. Of the respondents who sought no support, 11.3% reported this as being helpful.

**Conclusions:**

More than a third of secondary school students in this sample did not seek any help following self-harm. The majority of those not seeking help did not find this to be a helpful way of coping. Further work needs to determine effective ways of overcoming barriers to help-seeking among adolescents who self-harm and improving perceived helpfulness of the supports accessed.

## Introduction

Since the onset of the global pandemic there have been concerns about its impact on adolescent self-harm. Self-harm is defined as any act of intentional self-poisoning or self-injury, irrespective of motivation (1, 2). Evidence, however, suggests that presentations to hospital emergency departments due to self-harm in adolescents have decreased, including in England (3–5). Any reported change may reflect a proportionate change in the incidence of self-harm in the community. However, little is known about the incidence and prevalence of self-harm in the community in England since the onset of the pandemic. Furthermore, for those who have not presented to health services following self-harm during this period, little is known about the type(s) of support, if any, they have received.

Adolescent self-harm is a major public health problem (6) that is associated with numerous adverse health and social outcomes (7–9), including depression, substance misuse, poorer educational attainment, and dying by suicide (10). It has been suggested that in the years leading up to the pandemic, in England, the incidence of self-harm in adolescents has been increasing (11, 12). Self-harm can have a profound impact on the adolescents themselves, as well as their families and friends, health services, and the wider community (13, 14). Despite this, prior to the onset of the COVID-19 pandemic, approximately half of school-aged adolescents did not seek any support following an incident of self-harm (15), and only 1-in-8 presented to health services for medical treatment (16). Common reasons for this included the stigma and self-stigma associated with self-harm (17, 18), and a lack of knowledge about where to seek help (15). For those who do seek help, the previously published literature indicates that friends and family members are the most commonly reported sources of support (15). Help-seeking following self-harm is important because it represents an opportunity to offer individuals help and support which may mitigate the harmful impact of self-harm, irrespective of the motivation or intention associated with the behavior.

In this study, using data from a large sample of secondary school students in England surveyed after the onset of the pandemic, we aimed to (1) identify the prevalence of help-seeking among those with a history of self-harm; (2) examine the degree to which they perceived the support accessed as being helpful; and (3) identify the barriers to help-seeking behavior in students who did not access any support.

## Methods

The OxWell School Survey (19) is a cross-sectional survey examining the mental health and wellbeing of children and adolescents in England, conducted annually online since 2019. Students were invited to take part through their school using a parental opt-out process (20). In this study we report on the survey administered in 2020, which was completed either on school premises or from home due to partial school closures during the first COVID-19 UK national lockdown [described in greater detail elsewhere (20)].

### Participants

**Figure 1:**
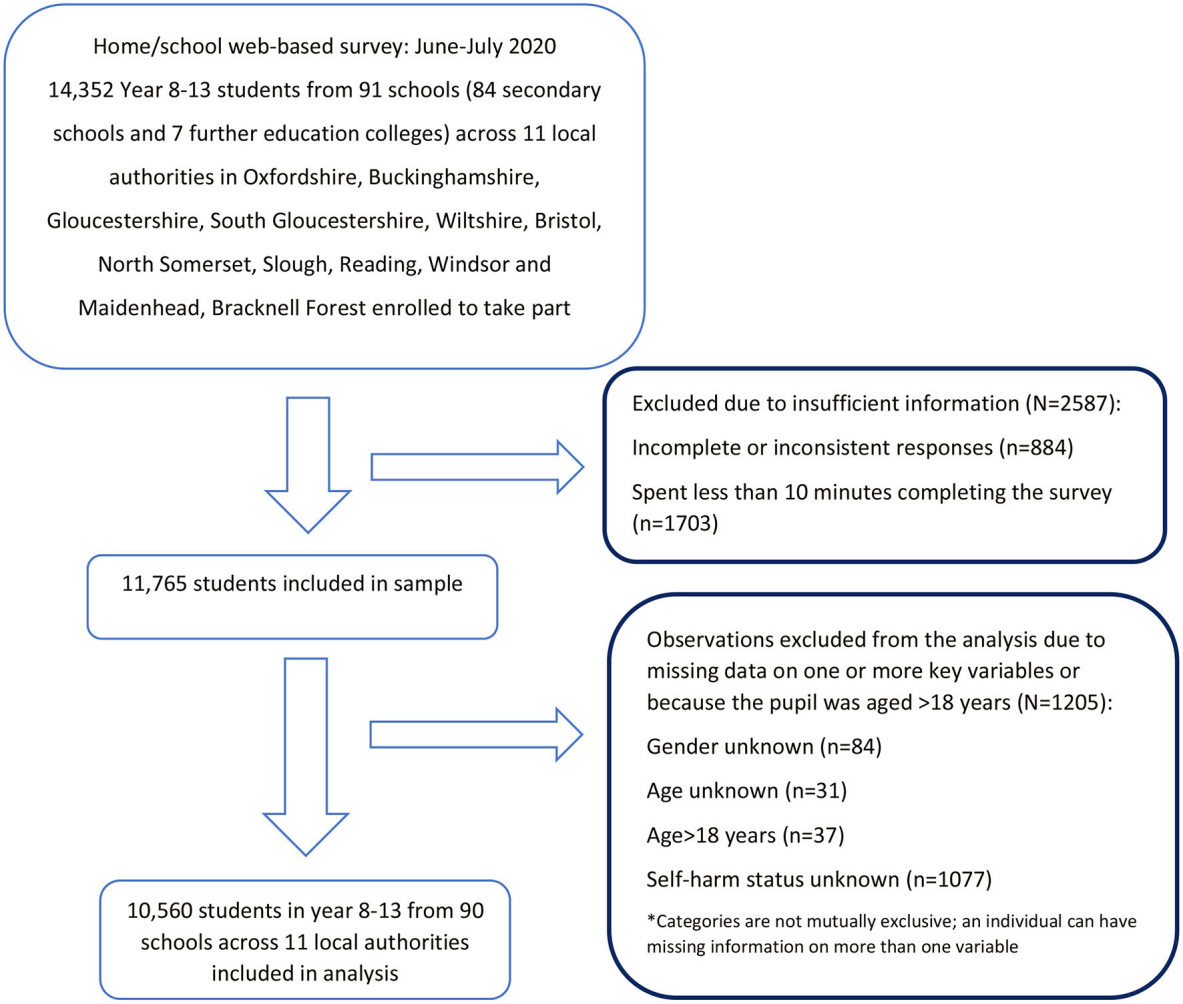
Sample selection process.

Schools were recruited through 11 local authorities (see Figure 1), and students were invited to participate by their school. Students attending school years 8–13 (aged 12–18 years) from secondary educational institutions in England—including all state-maintained schools, academies, and independent schools, as well as further education colleges (FECs) in the local authority areas—were eligible to participate.

### Measures

#### Demographics

Gender, year level (a proxy for age), whether the respondent/their parents were born in the UK (e.g. “Were you born in the UK?” *Yes/No/Rather not say*), and socioeconomic deprivation (two proxy measure: eligibility for free school meals or “school-assisted meals” and household food insecurity) were obtained *via* self-report. Being in receipt of free school meals is an official indicator of socioeconomic disadvantage in children and adolescents used in the UK. Information on school characteristics was obtained through linkage with data from the Office for National Statistics which are publicly available (21).

#### Self-Harm

Self-harm was defined as any act of non-fatal intentional self-poisoning or self-injury, irrespective of the nature or the motivation including degree of suicidal intent (2). Self-poisoning included the intentional ingestion of any drug where the amount exceeds that prescribed or the ingestion of non-ingestible substances, overdoses of “recreational drugs,” and severe alcohol intoxication where the individual intended to harm themselves. Self-injury was defined as any injury that was intentionally self-inflicted. Detailed questions relating to self-harm were based on those used in the Child and Adolescent Self-harm in Europe (CASE) study (22).

Lifetime self-harm was defined as intentional self-poisoning or self-injury which had occurred at any point prior to the survey. We used two questions to ascertain lifetime self-harm (Supplementary Table 1). For respondents who endorsed item *1* (“*Ever self-harmed*”) their free-text item (item 8) describing their act of self-harm was reviewed by two researchers (GG and ES) independently. They were classified with “lifetime self-harm” if their described act (item 8) met the study criteria (23). Past year self-harm was defined as intentional self-poisoning or self-injury which occurred in the 12 months prior to survey administration. Respondents who endorsed item *1* (“*Ever self-harmed)”*, had self-harmed within the past year (items *4*, “*Last self-injury*” and/or *7*, “*Last self-poisoned*”), and who described a valid method of self-harm (item *8*) were considered to have self-harmed in the past year. Self-harm in the past 6 months was defined as above, but had self-harmed within the past 6 months. Self-harm during lockdown was defined as intentional self-poisoning or self-injury which occurred between 23 March 2020 (i.e., the beginning of the first UK lockdown) and the date of survey administration (June-July 2020). Respondents who endorsed item *1* “*Ever self-harmed*” and item *2* “*Ever self-injured*” (from “*Once or twice'* to “*Daily*”) and item *3* “*Self-injured during lockdown*” (from “*Once or twice*” to “*Most days”*), or if they endorsed items *1* and *5* “*Ever self-poisoned*” (“*Yes*”) and *6* “*Self-poisoned during lockdown*” (“*Yes*”) were classified as having self-harmed during lockdown (provided their described act[s] of self-harm met the study criteria). All others were considered not to have self-harmed during lockdown (Supplementary Table 1).

Our method of self-harm ascertainment was contingent on the provision of information about the method of self-harm, in the form of free text. Some respondents who endorsed the item about self-harm did not provide further information on their act or provided information which was inconsistent with the study criteria (*n* = 999). The vast majority of those (*n* = 887, 88.8%) did not provide a free text response, 57 (5.7%) stated that they did not wish to provide further information, 18 (1.8%) described the reason or motivation for their act rather than their act, 7 (0.7%) denied self-harming, 4 (0.4%) described an act which was not consistent with self-harm (examples cannot be provided due to small numbers), 3 (0.3%) described the location and time of their self-harm, while the remaining 23 (2.3%) provided a range of other text options (e.g., a symbol).

Respondents who reported having self-harmed were asked whether or not they received support following self-harm from any of the following: Parent, step-parent, or carer; Brother or sister; Someone else in their family; Friends; GP (family doctor); Social Worker; School or college nurse/welfare staff; Psychologist or psychiatrist; Telephone helpline; Drop-in/advice center; Residential Warden; CAMHS (Child and Adolescent Mental Health Services); Website or online forum; or No-one, and the extent to which the support sought was perceived as helpful (response categories: Not helpful at all; Not helpful enough; Just about helpful enough; Quite helpful; Very helpful). Information about the reasons for not receiving support was sought from respondents who approached no-one for support. Respondents could select one or more of the following: Did not trust anyone; Did not want help; Didn't want to burden anyone else; Didn't want the stigma; Didn't know where to get help; Worried about it not being kept confidential; Scared/worried about what people might say; Other. Respondents who reported having self-harmed were further asked whether they required subsequent medical attention (i.e., whether or not they needed medical care therefore including only those who perceived a need for medical treatment), and the source of care they sought, including: Own first aid; Family-provided first aid; School nurse/first aid at school/college; Friends helped; GP (family doctor); Ambulance/paramedics; Hospital A&E / acute mental health provision; Hospital with overnight stay on ward; Other. Respondents were permitted to select multiple sources.

#### Mental Health Difficulties

For information about symptoms of depression and anxiety we used the Revised Children's Anxiety and Depression Scales (RCADS-25) (24, 25). We included participants who provided a response to at least 80% of the RCADS items. We derived standardized scores (t-scores) for depression and anxiety (Child Outcomes Research Consortium (26). We also created two binary groups with RCADS t-scores ≥ 70 indicating “probable depression/anxiety,” while a score <70 was categorized as “no depression/anxiety.” We further used the item: “Have you ever received any mental health support? Yes/No” to identify respondents with a history of mental health difficulties.

### Statistical Analysis

Respondents' characteristics, prevalence of self-harm, and indicators of care are presented as unweighted and weighted proportions with corresponding 95% confidence interval.

### Weights

Due to possible differences between the sample surveyed in the OxWell (19) survey and the target population (i.e., all those attending the identified schools), we applied post-stratification weights. Non-response may have arisen from multiple sources (i.e., differences in propensity to be involved by local authorities, schools and/or pupils). We calculated post-stratification weights to reduce possible non-response bias using raking and auxiliary information for a subset of demographics that could be matched with the UK Office for National Statistics (ONS) Census data for the participating counties. Weights were derived using regional census data including information on 1) type of school (independent vs. other i.e., state primary/secondary); 2) gender (male/female); 3) English as first language (we used a proxy of child and both parents born in UK); 4) age; and 5) Index of Multiple Deprivation (IMD; based on school address). The IMD is an official measure of deprivation of small geographical areas in England. It is a combined score from the following domains: income and employment, health and disability, education, skills and training, barriers to housing and services, living environment and crime. There are 32,844 small geographical areas across England which are ranked from 1 (most deprived) to 32,844 (least deprived). All analyses were conducted using Stata 14.2 (27).

## Results

A total of 10,560 students aged 12–18 years were enrolled in the study. Figure 1 shows the sample selection process.

The unweighted and weighted characteristics of the analytic sample are displayed in Table 1. Of the 10,560 respondents, 6,653 (53.7%) were female, and 5,429 (53.3%) were students attending years 8–9 (aged 12–14 years). Lifetime self-harm was more commonly reported by females (17.2%; 95% CI 16.2–18.1) than males (7.1%; 95% CI 6.3–8.0), and by older students than younger students (school years 12–13: 16.5%, 95% CI 14.8–18.2; years 10–11: 15.5%, 95% CI 14.3–16.8; years 8–9: 9.6%, 95% CI 8.8–10.4).

**Table 1:** Characteristics of the analytic sample, unweighted and weighted proportions with 95% confidence intervals, by gender.

	**Total**			**Males**			**Females**		
	***N***	**Unweighted %** **(95% CI)**	**Weighted %** **[95% CI]^**a**^**	***N***	**Unweighted %** **(95% CI)**	**Weighted % [95% CI]^**a**^**	***N***	**Unweighted %** **(95% CI)**	**Weighted** **% [95% CI]^**a**^**
*N* = 10,560				3,907	37.0 (36.1–37.9)	46.3 [45.3–47.3]	6,653	63.0 (62.1–63.9)	53.7 [52.7–54.7]
**Sociodemographic characteristics**									
School year									
Year 8–9 (age 12–14 years)	5,429	51.4 (50.1–52.4)	53.3 [52.3–54.3]	2,076	53.1 (51.6–54.7)	54.7 [53.1–56.3]	3,353	50.4 (49.2–51.6)	52.2 [50.9–53.3]
Year 10–11 (age 14–16 years)	3,291	31.2 (30.3–32.1)	29.5 [28.7–30.4]	1,111	28.4 (27.0–29.9)	27.3 [25.9–28.7]	2,180	32.8 (31.7–33.9)	31.5 [30.4–32.6]
Year 12–13 (age 16–18 years)	1,840	17.4 (16.7–18.2)	17.1 [16.4–17.9]	720	18.4 (17.2–19.7)	18.0 [16.8–19.2]	1,120	16.8 (16.0–17.8)	16.4 [15.5–17.3]
**Student born in the UK**									
Non–UK	1,307	12.4 (11.8–13.0)	17.5 [16.6–18.4]	509	13.0 (12.0–14.1)	18.3 [17.0–19.8]	798	12.0 (11.2–12.8)	16.8 [15.7–17.8]
UK	9,168	86.8 (86.2–87.5)	81.7 [80.8–82.6]	3,364	86.1 (85.0–87.2)	80.8 [79.4–82.2]	5,804	87.3 (86.4–88.0)	82.5 [81.4–83.5]
Unknown	85	0.8 (0.7–1.0)	0.8 [0.6–1.0]	34	0.9 (0.6–1.2)	0.9 [0.6–1.2]	51	0.8 (0.6–1.0)	0.8 [0.6–1.0]
**Parents born in the UK**									
Non–UK	3,887	36.8 (35.9–37.7)	40.6 [39.6–41.6]	1,483	38.0 (36.5–39.5)	41.7 [40.1–43.4]	2,404	36.1 (35.0–37.3)	39.6 [38.4–40.1]
UK	6,476	61.3 (60.4–62.3)	57.6 [56.6–58.6]	2,352	60.2 (58.7–61.7)	56.5 [54.9–58.1]	4,124	62.0 (61.8–63.1)	58.6 [57.3–59.8]
Unknown	197	1.9 (1.6–2.1)	1.8 [1.6–2.1]	72	1.8 (1.5–2.3)	1.8 [1.4–2.2]	125	1.9 (1.6–2.2)	1.9 [1.6–2.2]
**Free school meals**									
No	7,941	75.2 (74.4–76.0)	74.6 [73.7–75.4]	2,828	72.4 (71.0–73.8)	72.2 [70.7–73.6]	5,133	76.9 (75.8–77.9)	76.6 [75.6–77.7]
Yes	802	7.6 (7.1–8.1)	7.7 [7.2–8.2]	313	8.0 (7.2–8.9)	8.0 [7.1–8.9]	489	7.4 (6.8–8.0)	7.5 [6.8–8.1]
Not known	1,817	17.2 (16.5–17.9)	17.8 [17.0–18.6]	766	19.6 (18.4–20.9)	19.9 [18.6–21.2]	1,051	15.8 (14.9–16.7)	15.9 [15.1–16.9]
**Ever experienced food poverty**									
No	9,220	87.3 (86.7–87.9)	87.4 [86.7–88.0]	3,402	87.1 (86.0–88.1)	87.3 [86.1–88.3]	5,818	87.5 (86.6–88.2)	87.6 [86.7–88.4]
Yes^b^	938	8.9 (8.4–9.4)	8.8 [8.2–9.4]	338	8.7 (7.8–9.6)	8.6 [7.8–9.6]	600	9.0 (8.4–9.7)	8.9 [8.2–9.7]
Not known	402	3.8 (3.4–4.2)	3.8 [3.5–4.2]	167	4.3 (3.7–5.0)	4.2 [3.6–4.9]	235	3.5 (3.1–4.0)	3.5 [3.1–4.0]
**Mental health**									
Symptoms of depression (RCAD_D), mean (95% CI)^c^	10,465	50.6 (50.3–50.9)	49.7 [49.5–50.0]	3,858	45.9 (45.5–46.3)	45.8 [45.4–46.2]	6,607	53.2 (53.0–53.7)	53.1 [52.8–53.5]
Symptoms of anxiety (RCAD_A), mean (95% CI)^c^	10,465	49.8 (49.5–50.0)	49.1 [48.9–49.4]	3,858	46.0 (45.6–46.3)	45.9 [45.5–46.3]	6,607	52.0 (51.7–52.3)	51.8 [51.5–52.2]
**Ever received mental health support**									
No	7,895	74.8 (73.9–75.6)	76.3 [75.5–77.1]	3,194	81.8 (80.5–82.9)	82.0 [80.7–83.2]	4,701	70.7 (69.6–71.8)	71.4 [70.3–72.5]
Yes	2,588	24.5 (23.7–25.3)	23.0 [22.2–23.8]	688	17.6 (16.5–18.8)	17.4 [16.2–18.6]	1,900	28.6 (27.5–29.7)	27.8 [26.7–28.9]
Not known	77	0.7 (0.6–0.9)	0.7 [0.6–0.9]	25	0.6 (0.4–1.0)	0.6 [0.4–1.0]	52	0.8 (0.6–1.0)	0.8 [0.6–1.1]
**School characteristics**									
**Rural/urban**									
Rural	1,713	16.2 (15.5–16.9)	15.4 [14.8–16.2]	545	14.0 (12.9–15.1)	13.6 [12.6–14.7]	1,168	17.6 (16.7–18.5)	17.0 [16.1–18.0]
Urban	8,847	83.8 (83.1–84.5)	84.6 [83.9–85.3]	3,362	86.1 (84.9–87.1)	86.4 [85.3–87.5]	5,485	82.4 (81.5–83.3)	83.0 [82.1–83.9]
**Funding source**									
State funded	9,245	87.6 (86.9–88.2)	87.2 [86.6–87.9]	3,284	84.1 (82.9–85.2)	84.3 [83.1–85.4]	5,961	89.6 (88.9–90.3)	89.8 [89.0–90.5]
Independent	974	9.2 (8.7–9.8)	9.8 [9.2–10.4]	523	13.4 (12.4–14.5)	13.3 [12.2–14.4]	451	6.8 (6.2–7.4)	6.8 [6.2–7.4]
Not known (N/A)	341	3.2 (2.9–3.6)	3.0 [2.7–3.3]	100	2.6 (2.1–3.1)	2.4 [2.0–3.0]	241	3.6 (3.2–4.1)	3.5 [3.0–3.9]
**School type**									
Primary school	23	0.2 (0.15–0.3)	0.3 [0.2–0.4]	9	0.2 (0.1–0.4)	0.3 [0.1–0.5]	14	0.2 (0.1–0.4)	0.3 [0.2–0.5]
Secondary school	10,204	96.6 (96.3–97.0)	96.8 [96.5–97.1]	3,802	97.4 (96.8–97.8)	97.5 [96.9–97.9]	6,402	96.2 (95.7–96.7)	96.3 [95.9–96.8]
Further education	333	3.2 (2.8–3.5)	2.9 [2.6–3.2]	96	2.5 (2.0–3.0)	2.3 [1.9–2.9]	237	3.6 (3.1–4.0)	3.4 [3.0–3.9]
School type – gender									
% of mixed	7,423	70.3 (69.4–71.2)	70.8 [69.9–71.7]	2,885	73.8 (72.4–75.2)	73.9 [72.5–75.3]	4,538	68.2 (67.1–69.3)	68.1 [67.0–69.3]
**School index of multiple deprivation – quintiles**									
1st most deprived	497	4.7 (4.3–5.1)	4.8 [4.4–5.3]	119	3.1 (2.6–3.7)	3.4 [2.8–4.0]	378	5.7 (5.1–6.2)	6.1 [5.5–6.7]
2nd quintile	1,905	18.0 (17.3–18.8)	18.2 [17.5–19.0]	567	14.5 (13.4–15.7)	15.3 [14.2–16.6]	1,338	20.1 (19.1–21.0)	20.8 [19.8–21.8]
3rd quintile	1,008	9.6 (9.0–10.1)	9.5 [8.9–10.1]	408	10.4 (9.5–11.4)	10.1 [9.3–11.2]	600	9.0 (8.3–9.7)	8.9 [8.3–9.7]
4th quintile	1,944	18.4 (17.7–19.2)	18.4 [17.6–19.2]	797	20.4 (19.2–21.7)	20.1 [18.8–21.4]	1,147	17.2 (16.4–18.2)	17.1 [16.2–18.0]
5th least deprived	4,865	46.1 (45.1–47.0)	46.1 [45.1–47.1]	1,916	49.0 (47.5–50.6)	48.8 [47.2–50.4]	2,949	44.3 (43.2–45.6)	43.7 [42.5–45.0]
Not known	341	3.2 (2.9–3.6)	3.0 [2.7–3.3]	100	2.6 (2.1–3.1)	2.4 [2.0–3.0]	241	3.6 (3.2–4.1)	3.5 [3.0–3.9]

a *Weighted to account differences in the distribution of selected sociodemographic variables between the study sample and the target population*.

b *“Yes” includes those who reported having experienced food poverty from “once or twice” to “every day”*.

c *Excludes 95 (0.9%) observations where data were missing*.

*Clustering at the local authority, school or year group level did not inform calculation of the confidence intervals*.

Table 2 shows the unweighted and weighted prevalence of self-harm and the characteristics associated with help-seeking by gender.

**Table 2:** Prevalence of self–harm and care received, unadjusted and weighted proportions with 95% confidence intervals, by gender.

	**Total**			**Males**			**Females**		
	***N***	**Unweighted %** **(95% CI)**	**Weighted % [95% CI]^**a**^**	***N***	**Unweighted % (95% CI)**	**Weighted %** **[95% CI]^**a**^**	***N***	**Unweighted %** **(95% CI)**	**Weighted %** **[95% CI]^**a**^**
**Self–harm**									
Lifetime	1,457	13.8 (13.2–14.5)	12.5 [11.9–13.1]	285	7.3 (6.5–8.2)	7.1 [6.3–8.0]	1,172	17.6 (16.7–18.6)	17.2 [16.3–18.1]
Past year	1,133	10.7 (10.2–11.3)	9.6 [9.1–10.2]	206	5.3 (4.6–6.0)	5.2 [4.5–5.9]	927	13.9 (13.1–14.8)	13.5 [12.7–14.4]
Past six months	881	8.3 (7.8–8.9)	7.4 [7.0–7.9]	153	3.9 (3.4–4.6)	3.8 [3.2–4.4]	728	10.9 (10.2–11.7)	10.6 [9.8–11.3]
During 1^st^ UK lockdown	789	7.5 (7.0–8.0)	6.7 [6.2–7.2]	136	3.5 (3.0–4.1)	3.4 [2.8–4.0]	653	9.8 (9.1–10.6)	9.5 [8.9–10.3]
**Ever received support for self–harm, % yes by source of support (of 1,457)^b^**									
Parent, step–parent, or carer	369	25.3 (23.2–27.6)	25.0 [22.8–27.3]	71	24.9 (20.2–30.3)	24.2 [19.6–29.6]	298	25.4 (23.0–28.0)	25.2 [22.8–27.9]
Brother or sister	112	7.7 (6.4–9.2)	7.5 [6.3–9.1]	24	8.4 (5.7–12.3)	8.4 [5.6–12.3]	88	7.5 (6.1–9.2)	7.3 [5.9–8.9]
Someone else in your family	54	3.7 (2.9–4.8)	3.7 [2.8–4.8]	11	3.9 (2.2–6.8)	3.6 [2.0–6.4]	43	3.7 (2.7–4.9)	3.7 [2.7–5.0]
Friend(s)	533	36.6 (34.1–39.1)	35.9 [33.3–38.4]	92	32.3 (27.1–37.9)	31.4 [26.3–37.1]	441	37.6 (34.9–40.4)	37.5 [34.7–40.3]
GP (family doctor)	115	8.0 (6.6–9.4)	7.4 [6.2–8.9]	18	6.3 (4.0–9.8)	5.9 [3.7–9.1]	97	8.3 (6.8–10.0)	8.0 [6.6–9.7]
Social Worker	64	4.4 (3.5–5.6)	4.6 [3.6–5.9]	15	5.3 (3.2–8.6)	5.4 [3.2–8.8]	49	4.2 (3.2–5.5)	4.3 [3.2–5.7]
School or college nurse/welfare staff	208	14.3 (12.6–16.2)	13.6 [11.9–15.5]	23	8.1 (5.4–11.9)	8.0 [5.3–11.8]	185	15.8 (13.8–18.0)	15.6 [13.6–17.8]
Psychologist or psychiatrist	152	10.4 (8.8–9.1)	10.2 [8.7–11.9]	26	9.1 (6.3–13.1)	9.2 [6.3–13.3]	126	10.8 (9.1–12.7)	10.5 [8.9–12.4]
Telephone helpline	63	4.3 (3.4–5.5)	4.0 [3.1–5.1]	6	2.1 (1.0–4.6)	2.0 [0.9–4.3]	57	4.9 (3.8–6.3)	4.7 [3.6–6.1]
Drop–in/advice center	9	6.1 (3.2–11.8)	6.3 [3.2–12.6]	2	0.7 (0.2–2.8)	0.8 [0.2–3.4]	7	0.6 (0.3–1.3)	0.6 [0.3–11.8]
Residential Warden	3	0.2 (0.07–0.6)	0.2 [0.07–0.6]	1	0.4 (0.05–2.5)	0.3 [0.05–0.2]	2	0.2 (0.04–0.7)	0.2 [0.04–0.6]
CAMHS	184	12.6 (11.0–14.4)	12.1 [10.5–13.9]	27	9.5 (6.6–13.5)	9.6 [6.6–13.8]	157	13.4 (11.6–15.5)	12.9 [11.1–15.0]
Website or online forum	128	8.8 (7.4–10.4)	8.6 [7.2–10.2]	14	4.9 (2.9–8.1)	5.3 [3.1–8.8]	114	9.7 (8.2–11.6)	9.8 [8.2–11.7]
No–one ^**c**^	548	37.6 (35.2–40.1)	38.3 [35.7–40.9]	121	42.5 (36.8–48.3)	43.8 [38.1–49.8]	427	36.4 (33.7–39.2)	38.8 [36.0–41.7]
**How helpful was support received (of 1,457)**									
Not helpful at all	305	20.9 (18.9–23.2)	20.9 [18.9–23.2]	60	21.5 (16.7–26.2)	21.7 [17.2–27.1]	245	20.9 (18.7–23.3)	20.7 [18.4–23.1]
Not helpful enough	276	18.5 (17.0–21.0)	18.5 [16.6–20.7]	33	11.6 (8.3–15.6)	11.3 [8.1–15.5]	243	20.7 (18.5–23.2)	21.1 [18.8–23.6]
Just about helpful	329	22.6 (20.5–24.8)	22.8 [20.7–25.2]	74	26.0 (21.2–31.4)	25.6 [20.8–31.0]	255	21.8 (19.5–24.2)	21.9 [19.5–24.4]
Quite helpful	266	18.3 (16.4–20.3)	18.0 [16.0–20.0]	46	16.0 (12.3–20.9)	15.9 [12.1–20.7]	220	18.8 (16.6–21.1)	18.7 [16.5–21.1]
Very helpful	158	10.8 (9.4–12.6)	11.3 [9.7–13.2]	46	16.0 (12.3–20.9)	16.3 [12.3–21.2]	112	9.6 (8.0–11.4)	9.6 [8.0–11.5]
Not known	123	8.4 (7.1–10.0)	8.4 [7.0–10.0]	26	9.1 (6.3–13.1)	9.2 [6.3–13.3]	97	8.3 (6.8–10.0)	8.1 [6.6–9.8]
**Why did you not** ***receive*** **support? % yes (of 548 who did you not receive support)**^***c***^									
1. Did not trust anyone	226	41.2 (37.2–45.4)	40.0 [35.9–44.3]	35	28.9 (21.5–37.7)	27.7 [20.5–36.4]	191	44.7 (40.1–49.5)	45.2 [40.4–50.1]
2. Did not want help	320	58.4 (54.2–62.5)	59.3 [55.0–63.5]	82	67.8 (58.9–75.5)	66.9 [57.8–74.9]	238	55.7 (51.0–60.4)	56.1 [51.3–61.8]
3. Didn't want to burden anyone else	303	55.3 (51.1–59.4)	54.5 [50.2–58.8]	59	48.8 (39.9–57.7)	48.2 [39.2–57.2]	244	57.1 (52.4–61.8)	57.2 [52.4–61.9]
4. Didn't want the stigma	143	26.1 (22.6–30.0)	25.0 [21.5–28.9]	26	21.5 (15.0–29.8)	20.5 [14.2–28.6]	117	27.4 (23.4–31.8)	27.0 [22.9–31.4]
5. Didn't know where to get help	67	12.2 (9.7–15.3)	11.5 [9.1–14.4]	7	5.7 (2.8–11.7)	5.3 [2.5–10.7]	60	14.1 (11.1–17.8)	14.1 [11.1–17.8]
6. Worried about it not being kept confidential	232	42.3 (38.3–46.5)	41.4 [37.2–45.7]	41	33.9 (26.0–42.8)	32.9 [25.0–41.8]	191	44.7 (40.1–49.5)	45.0 [40.3–49.9]
7. Scared/worried about what people might say	270	49.3 (45.1–53.5)	48.0 [43.6–52.3]	49	40.5 (32.1–49.5)	39.7 [31.3–48.9]	221	51.8 (47.0–56.5)	51.4 [46.6–56.2]
8. Other	106	19.3 (16.2–22.9)	19.3 [16.1–23.0]	20	16.5 (10.9–24.3)	17.2 [11.3–25.4]	86	20.2 (16.6–24.2)	20.5 [16.9–24.5]
**Needed treatment? % yes (of 1,457)^b^**									
My own first aid	779	53.5 (50.9–56.0)	52.5 [49.8–55.1]	113	39.7 (34.1–45.5)	39.8 [34.2–45.7]	666	56.8 (54.0–59.6)	57.0 [54.1–59.8]
Family–provided first aid	88	6.0 (4.9–7.4)	5.6 [4.6–6.9]	8	2.8 (1.4–5.5)	2.6 [1.3–5.3]	80	6.8 (5.5–8.4)	6.7 [5.4–8.3]
School nurse/first aid at school/college	59	4.0 (3.2–5.2)	3.9 [3.0–5.0]	7	2.5 (1.2–5.1)	2.3 [1.1–4.7]	52	4.4 (3.4–5.8)	4.5 [3.4–5.9]
Friends helped me	126	8.7 (7.3–10.2)	8.5 [7.2–10.1]	19	6.7 (4.3–10.2)	6.2 [4.0–9.5]	107	9.1 (7.6–10.9)	9.4 [7.8–11.3]
GP (family doctor)	42	2.9 (2.1–3.9)	2.7 [2.0–3.7]	6	2.1 (1.0–4.6)	2.0 [0.1–4.2]	36	3.1 (2.2–4.2)	3.0 [2.2–4.1]
Ambulance/paramedics	25	1.7 (1.2–2.5)	1.6 [1.1–2.3]	2	0.7 (0.2–2.8)	0.8 [0.2–0.9]	23	2.0 (1.3–2.9)	1.8 [1.2–2.8]
Hospital A&E / acute mental health provision	52	3.6 (2.7–4.7)	3.4 [2.6–4.5]	12	4.2 (2.4–7.3)	3.9 [2.2–6.8]	40	3.4 (2.5–4.6)	3.3 [2.4–4.4]
Hospital with overnight stay on ward	43	3.0 (2.2–4.0)	2.8 [2.1–3.8]	8	2.8 (1.4–5.5)	3.0 [1.5–5.9]	35	3.0 (2.2–4.1)	2.8 [2.0–3.9]
Other	126	8.7 (7.3–10.2)	8.5 [7.2–10.1]	25	8.8 (6.0–12.7)	8.7 [5.9–12.6]	101	8.6 (7.1–10.4)	8.4 [7.0–10.2]

a *Weighted to account for differences in the distribution of selected sociodemographic variables between the study sample and the target population*.

b *Can include more than one response*.

c *Includes only those who sought no support*.

*Clustering at the local authority, school or year group level did not inform calculation of the confidence intervals*.

1,457 respondents (12.5%) reported a lifetime history of self-harm, and past-year self-harm was reported by 1,133 participants (9.6%). Self-harm that had occurred during the period of lockdown (between 23 March 2020 and when respondents completed the survey, in June-July 2020) was reported by 789 (6.7%) respondents; 653 (9.5%) females and 136 (3.4%) males. More than one in three respondents who self-harmed (38.3%) reported not receiving any support.

Of the 1,457 respondents who had *ever* self-harmed, the highest proportion reported accessing informal sources of support (friends: 35.9%; parents or carers: 25.0%; sibling: 7.5%). Considerably fewer adolescents reported accessing clinical services (child and adolescent mental health services [CAMHS]: 12.1%; psychologist or psychiatrist: 10.2%; general practitioner [GP]: 7.4%). Just 8.6% accessed support through a website or online forum and 4.0% received support from a telephone helpline. Most respondents who believed they required medical treatment following self-harm reported using first aid provided by themselves (52.5%), friends (8.5%), or family (5.6%), whilst the proportion of respondents needing medical treatment who presented to medical services was <5% (Table 2).

**Figure 2:**
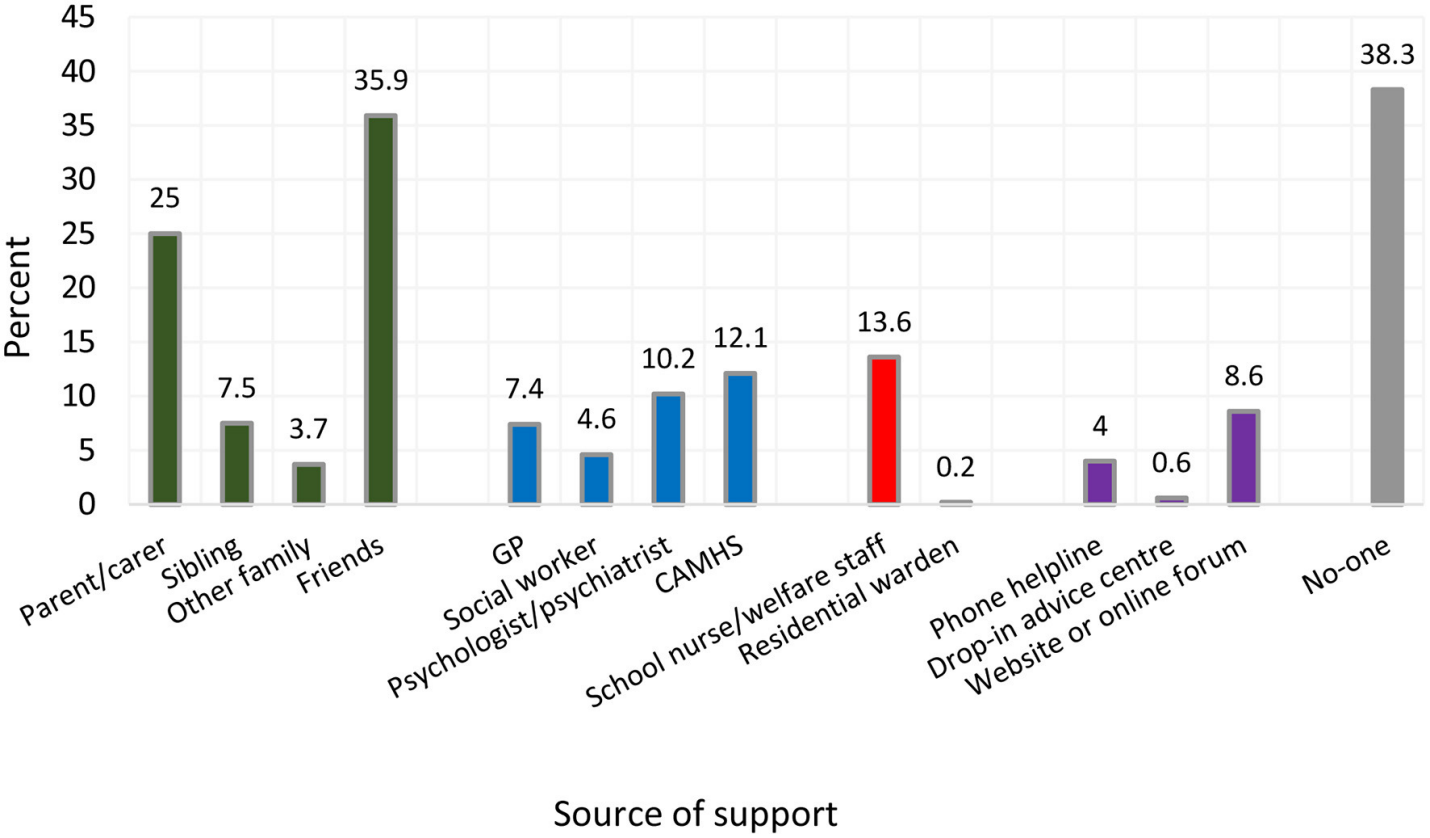
Source of support accessed following self–harm, weighted proportions^a^. ^a^Weighted to account for differences in the distribution of selected sociodemographic variables between the study sample and the target population.

**Figure 3:**
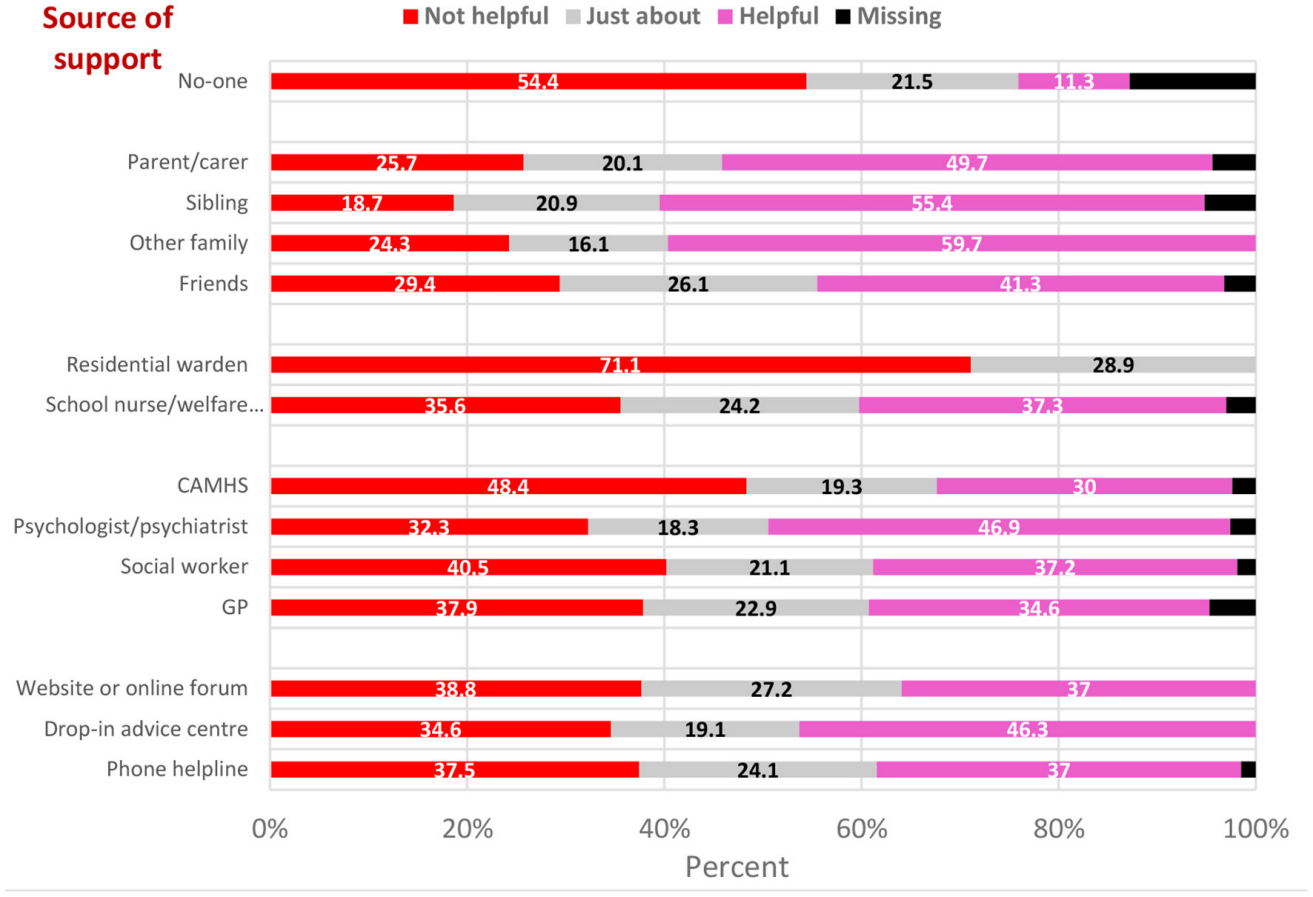
Level of satisfaction with support received by source of support, weighted proportions^a^. ^a^Weighted to account for differences in the distribution of selected sociodemographic variables between the study sample and the target population.

Informal sources of support were reported to be the most helpful of all sources (other family members: 59.7%; sibling: 55.4%; parents or carers: 49.7%). Most respondents (54.4%) who received no support reported that they found this unhelpful. Of the clinical services accessed, the proportion of respondents who reported finding them helpful ranged from 30.0% (CAMHS) to 46.9% (psychologist/psychiatrist; Table 2, Figures 2, 3).

Of the respondents who did not access support, more than half (59.3%) reported that they did not want help and 55.3% reported that they did not wish to burden others. Other widely cited reasons for not seeking support included concern about others' opinions (48.0%) and the possibility of a breach of confidentiality (41.4%). One in four (25.0%) reported that they did not access support because they did not want the stigma associated with self-harm, and 11.5% reported not accessing support because they did not know where to find it (Table 2). Of those who did not access support, one in nine (11.3%) reported this as helpful. Respondents who reported not accessing any support were more likely to have parents born outside the UK, more likely to attend male-only schools, and less likely to be eligible to receive free school meals (Supplementary Table 2).

We compared the patterns of supports accessed by adolescents who self–harmed during lockdown to supports accessed prior to the pandemic. Of the adolescents who reported self–harming during lockdown (*n* = 789, 6.7% of adolescents surveyed), most reported accessing informal sources of support [friends: 36.5% (95% CI 33.1–40.0); parents or carers: 23.6% (95% CI 20.7–26.7); sibling: 7.0% (95% CI 5.3–9.1)]. Smaller numbers accessed clinical services [(CAMHS: 14.5% (95% CI 12.2–17.1); psychologist or psychiatrist: 11.1% (95% CI 9.1–13.5); GP: 8.2% (95% CI 6.5–10.2)]. Just 10.2% (95% CI 8.2–12.5) accessed support through a website or online forum and 5.6% (95% CI 4.2–7.4) received support from a telephone helpline. 38.2% (95% CI 34.7–41.8) accessed no support. The overall pattern was similar in adolescents who self–harmed prior to the pandemic (*n* = 668, 5.8% of adolescents surveyed) with most accessing informal sources [friends: 35.1% (95% CI 31.5–38.9), parents or carers: 26.6% (95% CI 23.3–30.2) and sibling: 8.1% (95% CI 6.2–10.5)]. The number of adolescents accessing all other types of support was markedly smaller [(CAMHS: 9.2% (95% CI 7.2–11.8); psychologist or psychiatrist: 9.1% (95% CI 7.2–11.6); GP: 6.6% (95% CI 4.9–8.7)]. 6.8% (95% CI 5.1–9.1) accessed support through a website or online forum and 2.1% (95% CI 1.3–3.5) received support from a telephone helpline while 38.4% (95% CI 34.6–42.3) accessed no support. Nevertheless, the proportion of adolescents who received support through CAMHS was comparatively lower in adolescents who self–harmed before the pandemic [9.2% (95% CI 7.2–11.8)] relative to those who reported self–harm since its onset [14.5% (95% CI 12.2–17.1)]. Similarly, the proportion of adolescents who accessed support through a website or online forum was somewhat lower in the pre–pandemic [6.8% (95% CI 5.1–9.1)] than since its onset 10.2% (95% CI 8.2–12.5)].

## Discussion

In our sample of 10,560 secondary school students aged 12–18 years, 12.5% reported lifetime self–harm and 6.7% reported self–harming during the previous three months (the period coinciding with the UK's first national COVID−19 lockdown in 2020). We observed marked differences in the reported utilization of various sources of support and the degree to which these were perceived as helpful. Accessing informal sources of support and not accessing any support were the most frequent responses. Although no formal tests for significance were conducted, many of the confidence intervals overlapped heavily and, on this basis, there appeared to be no gender differences in any categories.

### Informal Support

Of all available sources of support, informal sources (Parent, step–parent, or carer; Brother or sister; Someone else in their family; Friends) were accessed by the highest proportion of respondents. These were also reported to be the most helpful of all options listed, with 60% and 50% of respondents reporting that seeking help from family members and parents/ caregivers, respectively, was helpful. In the context of the national COVID−19 lockdown that coincided with our data collection period, informal sources of support may have been more readily accessible than clinical or school–based support services. However, this pattern was seen whether adolescents self–harmed prior to or since the onset of the pandemic. Furthermore, a 2014 systematic review of adolescent help–seeking behavior following self–harm (15) also reported that young people primarily turn to friends and family members for support following an act of self–harm, suggesting that COVID−19 may not have been a unique contributing factor to this finding.

### Formal Clinical Support

Fewer than 1–in−8 respondents with a history of self–harm reported accessing support from mental health services, a psychiatrist, psychologist, GP, or social worker. Furthermore, clinical services were perceived to be less helpful than informal sources of support such as friends and family members. During the first UK lockdown, there was a significant reduction in the number of referrals to mental health services, including to CAMHS (28). This was driven partly by a reduced healthcare workforce due to sickness and self–isolation, and by substantial changes in service configuration and accessibility which are likely to have influenced our findings. The low prevalence of help–seeking observed in our study supports previous research in this area prior to the onset of the COVID−19 pandemic (16).

### Online and Phone–Based Support

Telephone helplines and web–based forums—freely available and possibly more prominent in the COVID−19 lockdowns—were accessed by the lowest proportion of respondents (4–8%). This pattern was observed in adolescents who self–harmed prior to and since the onset of the pandemic. These sources were also rated as the least helpful of all available sources of support, with just over a third reporting that they found these services helpful. Our finding that adolescents did not access support from readily available, anonymous, cost–neutral sources—even in the relative absence of more formal support options due to the COVID−19 lockdown—requires further investigation. Many clinical services, along with the wider public health and research agenda, have placed considerable emphasis on developing virtual resources to support mental health (29, 30). Yet, despite being forced to spend more time in the virtual environment due to education shifting online for most students, our findings suggest that young people have not turned to such resources in times of acute distress. This may reflect a lack of awareness of these resources, or the belief they may not be helpful (or both) and highlight the importance of ensuring that if online resources are developed, they are informed by the young people themselves. Such work may be of benefit in two ways here: first, it may help us understand how to make information about available support(s) more accessible to the relevant users (e.g., *via* social media platforms). Second, if young people are aware of existing sources of support but perceive them to be unhelpful, further work may identify alternative approaches they might find potentially helpful.

### No Support Accessed

Approximately two in five respondents who reported a history of self–harm did not access any sources of support. The most common reasons cited for not accessing support were 1) not wanting help; 2) not wanting to burden anybody; and 3) being scared or worried about what others might say. Other reasons included not trusting anyone and not knowing where to access support, both of which were endorsed by a higher proportion of females than males. Importantly, more than half of this group reported that not accessing any support following self–harm was unhelpful and only one in nine stated that it was helpful. Furthermore, many of the reasons most frequently endorsed by the respondents for not seeking help (e.g., stigma, feeling burdensome, and others' opinions) are related to shame and fear. This suggests that some young people who self–harm would welcome either formal or informal support to better manage their self–harm and/or the distress associated with it, yet, paradoxically, they are not accessing such support. In light of this unmet need, more work is required to understand the reasons why the young people did not seek help.

Our finding that 38% of respondents did not access any support following self–harm expands on previous review findings (15), which noted that up to one half of adolescents who self–harm do not seek help afterwards. They also support previous UK–based research indicating that most self–harm among young adolescents does not come to the attention of clinical services (6), and the common reasons provided for not accessing help following self–harm (15, 31, 32). More work could be carried out within schools and other relevant settings to address barriers such as concerns about privacy, availability of support and stigma surrounding mental health difficulties. Further work is also needed to better understand the response of 60% of adolescents who did not receive support because they did not want help, and the extent to which they did not access any support because they perceived the support available to be unhelpful.

### Medical Intervention Following Self–Harm

All respondents who reported self–harm were asked if they required medical treatment after their most recent episode of self–harm. Of these, less than one in twenty reported accessing help from an ambulance, GP, or hospital emergency department. Rather, most applied their own first aid or received assistance from friends or family members. A larger proportion of females than males reported applying their own first aid and receiving help from a family member. Adolescent self–harm may signal the occurrence of other risk behaviors posing additional hazards for young people (7), including increased risk of premature death (14, 33). Although self–harm varies substantially in terms of medical severity, it is concerning that most young people who thought they needed medical intervention did not seek appropriate help. There are likely to be high levels of untreated morbidity and distress among this population and facilitating the help–seeking of this difficult–to–reach group of young people should be considered an urgent priority. Alongside wider, population–based strategies to reduce mental health–related stigma (34), young people may benefit from decisional support aids addressing a variety of psychosocial and physical needs (35). Our findings suggest that parents and friends need more effective methods to help those who have self–harmed and to facilitate help–seeking where appropriate, and services and policymakers need to ensure that these supports are acceptable and accessible to adolescents in a timely manner. Ideally, all young people should have access to evidence–based guidance to help them manage their own self–harm (if applicable), and/or to offer support to their friends who may be engaging in self–harm. Similarly, it is important to determine which resources would be most helpful for parent and carers as well as school staff.

Consideration must also be given to the optimal social scaffolding that will support students' ongoing emotional development and minimize the risk of self–harm, and to promote protective and enabling relationships with families, schools, communities, and peers (36).

Furthermore, schools may be an important setting to address self–harm. There is some evidence of beneficial effects of several school–based programmes addressing self–harm in adolescence. Such programmes include the Saving and Empowering Young Lives in Europe (SEYLE) (37) and the Good Behavior Game (38). The Signs of Suicide (SOS) prevention programme has also reported some beneficial effect in terms of reduction in self–harm behavior (39), although the results of this programme has not been replicated in a UK population. An earlier UK school–based qualitative analysis provided some evidence about the benefit of peer support (16). Other possible approaches include developing resources tailored to the needs of specific groups (as discussed above) although their impact has not been evaluated.

### Limitations and Strengths

Our findings should be considered in light of some potential limitations. First, the disclosure of self–harm remains highly stigmatizing among many young people (17) and it is therefore possible that we under–ascertained the prevalence of self–harm in our sample (40). However, we did not collect identifiers such as name, address, or postcode to preserve students' anonymity and to encourage accurate responses to questions around self–harm and other sensitive items relevant to mental health. Second, our method of self–harm ascertainment was contingent on the provision of information about the method of self–harm in the form of a free text. Some respondents who reported self–harming did not provide further information on their acts and therefore were not classified as having self–harmed. Our estimates of the prevalence of self–harm are therefore likely to be conservative especially as a distressing memory associated with the self–harm incident might make it less likely for a student to describe what they had done. Third, our sample included students enrolled in and actively attending school; as such, we did not capture the experiences of young people who have disengaged from education and who are at increased risk of experiencing poor mental health (41). Fourth, our data were collected in the context of partial school closures resulting from a national COVID−19 lockdown and not in a standard educational setting. This may have influenced our findings. Our quantitative findings would have been strengthened by additional qualitative data to better understand the lived experience of adolescents during this time, and how they perceived access to support and services during lockdown (42). Fifth, we did not include any measure of non–binary gender identification. As this is associated with an increased prevalence of self–harm (43), this may have further contributed to an under–ascertainment of self–harm. Finally, the study did not allow for a clear separation in support sought during different timeframes. Our items about support sought after self–harm were phrased to capture support sought at any point in time although we have shown that the patterns of support accessed were similar in adolescents who only reported self–harm prior to the pandemic and those who self–harmed during the first lockdown. Our study has several strengths. Data were collected from a large sample of students, attending 90 schools across four demographically and socio–economically disparate counties in England. Data were also collected during a national COVID−19 lockdown incorporating partial school closures, providing a contemporaneous snapshot of adolescent self–harm during this unique period.

## Conclusions

We found that two in five secondary school students who have self–harmed did not access any sources of support. Most implied that this was not a helpful approach, thus highlighting a group likely to benefit from more accessible and appropriate support options following self–harm. In the context of the COVID−19 pandemic's documented adverse impacts on the mental health of adolescents (44, 45), the imperative to effectively identify and support adolescents engaging in (or at increased risk of) self–harm has never been more urgent. Identifying those young people who self–harm but do not subsequently access support (who may be at increased risk of poor outcomes) should be considered a particularly high priority. Young people primarily turn to friends and family members for support following an act of self–harm. These friends and family members may experience distress because of their concern for the young person's well–being and also because they may not feel they have the skills and knowledge on how best to support the young person who has self–harmed, highlighting an important group of individuals who may benefit from guidance and support themselves.

## Data Availability Statement

All authors that were Oxford-based members of the OxWell research team at the time of data analysis (GG, KM, and MF) had full access to all the data in the study and accepted responsibility to submit for publication. Fully anonymised extracts of the data can be provided to academic research collaborators upon reasonable request, following a review process by the research team to ensure uses of the data fall under the remit of the intended purposes set out in the privacy information and to prevent duplication of analyses. The data are not publicly available due to ethical and information governance restrictions. The full list of questions and other details are available on a project-specific ‘OxWell' OSF website along with publication of the study protocol (see: https://osf.io/sekhr/). Full data dictionaries can be made available upon approval for access to data extracts.

## Ethics Statement

Pupils under 16 years gave active assent to participate; those 16 years and over consented to the study. The study received ethical approval (Ref R62366/RE0010) from the University of Oxford Medical Sciences Division Research Ethics Committee (MSDREC).

## Author Contributions

MF and KM conceived the OxWell School Survey and obtained funds. All authors worked on conceptualization and methodology. GG designed and performed the analyses. RB wrote the first draft of the manuscript. All authors critically reviewed the manuscript and approved the final version for submission.

## Funding

This research was funded by the National Institute for Health Research (NIHR) Applied Research Collaboration Oxford and Thames Valley at Oxford Health NHS Foundation Trust (MF), the NIHR Oxford Health Biomedical Research Centre (BRC-1215-20005 to KM), an MRC Mental Health Data Pathfinder award (MC_PC_17215) to the University of Oxford, and the Westminster Foundation. KH is a member of the National Suicide Prevention Strategy for England Advisory Group and is funded by Oxford Health NHS Foundation Trust. PM is part-funded by the NIHR Biomedical Research Center at University Hospitals Bristol and Weston NHS Foundation Trust and the University of Bristol and also receives salary support from Avon & Wiltshire Mental Health Partnership NHS Trust. RB is funded by an Australian National Health and Medical Research Council (NHMRC) Emerging Leadership-2 Investigator Grant (GNT2008073). The funders played no role in the writing of the manuscript, or the decision to submit for publication. The views expressed in this publication are those of the author(s) and not necessarily those of the NIHR, the MRC, or the Department of Health and Social Care. GG is funded by the Department of Health and Social Care, UK.

## Conflict of Interest

The authors declare that the research was conducted in the absence of any commercial or financial relationships that could be construed as a potential conflict of interest.

## Publisher's Note

All claims expressed in this article are solely those of the authors and do not necessarily represent those of their affiliated organizations, or those of the publisher, the editors and the reviewers. Any product that may be evaluated in this article, or claim that may be made by its manufacturer, is not guaranteed or endorsed by the publisher.
