# Supplementary material for: Utilization and Acceptability of Formal and Informal Support for Adolescents Following Self-Harm Before and During the First COVID-19 Lockdown: Results From a Large-Scale English Schools Survey
Source: Front Psychiatry. 2022 Jun 24;13:881248. doi: 10.3389/fpsyt.2022.881248 (PMC9263724; doi:10.3389/fpsyt.2022.881248)
Supplement: Supplementary file 1 [file Table_1.DOCX]

**RESULTS**

**Figure 1: Sample selection process**

Home/school web-based survey: June-July 2020

14,352 Year 8-13 students from 91 schools (84 secondary schools and 7 further education colleges) across 11 local authorities in Oxfordshire, Buckinghamshire, Gloucestershire, South Gloucestershire, Wiltshire, Bristol, North Somerset, Slough, Reading, Windsor and Maidenhead, Bracknell Forest enrolled to take part

Excluded due to insufficient information (N=2587):

Incomplete or inconsistent responses (n=884)

Spent less than 10 minutes completing the survey (n=1703)

11,765 students included in sample

Observations excluded from the analysis due to missing data on one or more key variables or because the pupil was aged >18 years (N=1205):

Gender unknown (n=84)

Age unknown (n=31)

Age>18 years (n=37)

Self-harm status unknown (n=1077)

*Categories are not mutually exclusive; an individual can have missing information on more than one variable

10,560 students in year 8-13 from 90 schools across 11 local authorities included in analysis

**Table 1: Characteristics of the analytic sample, unweighted and weighted proportions with 95% confidence intervals, by gender**

|  | Total | | | Males | | | Females | | |
| --- | --- | --- | --- | --- | --- | --- | --- | --- | --- |
|  | N | Unweighted % (95% CI) | Weighted % [95% CI]^a^ | N | Unweighted % (95% CI) | Weighted % [95% CI]^a^ | N | Unweighted % (95% CI) | Weighted % [95% CI]^a^ |
| N=10,560 | | |  | 3,907 | 37.0 (36.1-37.9) | 46.3 [45.3-47.3] | 6,653 | 63.0 (62.1-63.9) | 53.7 [52.7-54.7] |
| Sociodemographic characteristics | | |  |  |  |  |  |  |  |
| School year |  |  |  |  |  |  |  |  |  |
| Year 8-9 (age 12-14 years) | 5,429 | 51.4 (50.1-52.4) | 53.3 [52.3-54.3] | 2,076 | 53.1 (51.6-54.7) | 54.7 [53.1-56.3] | 3,353 | 50.4 (49.2-51.6) | 52.2 [50.9-53.3] |
| Year 10-11 (age 14-16 years) | 3,291 | 31.2 (30.3-32.1) | 29.5 [28.7-30.4] | 1,111 | 28.4 (27.0-29.9) | 27.3 [25.9-28.7] | 2,180 | 32.8 (31.7-33.9) | 31.5 [30.4-32.6] |
| Year 12-13 (age 16-18 years) | 1,840 | 17.4 (16.7-18.2) | 17.1 [16.4-17.9] | 720 | 18.4 (17.2-19.7) | 18.0 [16.8-19.2] | 1,120 | 16.8 (16.0-17.8) | 16.4 [15.5-17.3] |
| Student born in the UK | | | |  |  |  |  |  |  |
| Non-UK | 1,307 | 12.4 (11.8-13.0) | 17.5 [16.6-18.4] | 509 | 13.0 (12.0-14.1) | 18.3 [17.0-19.8] | 798 | 12.0 (11.2-12.8) | 16.8 [15.7-17.8] |
| UK | 9,168 | 86.8 (86.2-87.5) | 81.7 [80.8-82.6] | 3,364 | 86.1 (85.0-87.2) | 80.8 [79.4-82.2] | 5,804 | 87.3 (86.4-88.0) | 82.5 [81.4-83.5] |
| Unknown | 85 | 0.8 (0.7-1.0) | 0.8 [0.6-1.0] | 34 | 0.9 (0.6-1.2) | 0.9 [0.6-1.2] | 51 | 0.8 (0.6-1.0) | 0.8 [0.6-1.0] |
| Parents born in the UK | | | |  |  |  |  |  |  |
| Non-UK | 3,887 | 36.8 (35.9-37.7) | 40.6 [39.6-41.6] | 1,483 | 38.0 (36.5-39.5) | 41.7 [40.1-43.4] | 2,404 | 36.1 (35.0-37.3) | 39.6 [38.4-40.1] |
| UK | 6,476 | 61.3 (60.4-62.3) | 57.6 [56.6-58.6] | 2,352 | 60.2 (58.7-61.7) | 56.5 [54.9-58.1] | 4,124 | 62.0 (61.8-63.1) | 58.6 [57.3-59.8] |
| Unknown | 197 | 1.9 (1.6-2.1) | 1.8 [1.6-2.1] | 72 | 1.8 (1.5-2.3) | 1.8 [1.4-2.2] | 125 | 1.9 (1.6-2.2) | 1.9 [1.6-2.2] |
| Free school meals |  |  |  |  |  |  |  |  |  |
| No | 7,941 | 75.2 (74.4-76.0) | 74.6 [73.7-75.4] | 2,828 | 72.4 (71.0-73.8) | 72.2 [70.7-73.6] | 5,133 | 76.9 (75.8-77.9) | 76.6 [75.6-77.7] |
| Yes | 802 | 7.6 (7.1-8.1) | 7.7 [7.2-8.2] | 313 | 8.0 (7.2-8.9) | 8.0 [7.1-8.9] | 489 | 7.4 (6.8-8.0) | 7.5 [6.8-8.1] |
| Not known | 1,817 | 17.2 (16.5-17.9) | 17.8 [17.0-18.6] | 766 | 19.6 (18.4-20.9) | 19.9 [18.6-21.2] | 1,051 | 15.8 (14.9-16.7) | 15.9 [15.1-16.9] |
| Ever experienced food poverty |  |  |  |  |  |  |  |  |  |
| No | 9,220 | 87.3 (86.7-87.9) | 87.4 [86.7-88.0] | 3,402 | 87.1 (86.0-88.1) | 87.3 [86.1-88.3] | 5,818 | 87.5 (86.6-88.2) | 87.6 [86.7-88.4] |
| Yes^b^ | 938 | 8.9 (8.4-9.4) | 8.8 [8.2-9.4] | 338 | 8.7 (7.8-9.6) | 8.6 [7.8-9.6] | 600 | 9.0 (8.4-9.7) | 8.9 [8.2-9.7] |
| Not known | 402 | 3.8 (3.4-4.2) | 3.8 [3.5-4.2] | 167 | 4.3 (3.7-5.0) | 4.2 [3.6-4.9] | 235 | 3.5 (3.1-4.0) | 3.5 [3.1-4.0] |
| Mental health |  |  |  |  |  |  |  |  |  |
| Symptoms of depression (RCAD_D), mean (95% CI)^c^ | 10,465 | 50.6 (50.3-50.9) | 49.7 [49.5-50.0] | 3,858 | 45.9 (45.5-46.3) | 45.8 [45.4-46.2] | 6,607 | 53.2 (53.0-53.7) | 53.1 [52.8-53.5] |
| Symptoms of anxiety (RCAD_A), mean (95% CI)^c^ | 10,465 | 49.8 (49.5-50.0) | 49.1 [48.9-49.4] | 3,858 | 46.0 (45.6-46.3) | 45.9 [45.5-46.3] | 6,607 | 52.0 (51.7-52.3) | 51.8 [51.5-52.2] |
| Ever received mental health support | | | |  |  |  |  |  |  |
| No | 7,895 | 74.8 (73.9-75.6) | 76.3 [75.5-77.1] | 3,194 | 81.8 (80.5-82.9) | 82.0 [80.7-83.2] | 4,701 | 70.7 (69.6-71.8) | 71.4 [70.3-72.5] |
| Yes | 2,588 | 24.5 (23.7-25.3) | 23.0 [22.2-23.8] | 688 | 17.6 (16.5-18.8) | 17.4 [16.2-18.6] | 1,900 | 28.6 (27.5-29.7) | 27.8 [26.7-28.9] |
| Not known | 77 | 0.7 (0.6-0.9) | 0.7 [0.6-0.9] | 25 | 0.6 (0.4-1.0) | 0.6 [0.4-1.0] | 52 | 0.8 (0.6-1.0) | 0.8 [0.6-1.1] |
| School characteristics | | |  |  |  |  |  |  |  |
| Rural/urban |  |  |  |  |  |  |  |  |  |
| Rural | 1,713 | 16.2 (15.5-16.9) | 15.4 [14.8-16.2] | 545 | 14.0 (12.9-15.1) | 13.6 [12.6-14.7] | 1,168 | 17.6 (16.7-18.5) | 17.0 [16.1-18.0] |
| Urban | 8,847 | 83.8 (83.1-84.5) | 84.6 [83.9-85.3] | 3,362 | 86.1 (84.9-87.1) | 86.4 [85.3-87.5] | 5,485 | 82.4 (81.5-83.3) | 83.0 [82.1-83.9] |
| Funding source |  |  |  |  |  |  |  |  |  |
| State funded | 9,245 | 87.6 (86.9-88.2) | 87.2 [86.6-87.9] | 3,284 | 84.1 (82.9-85.2) | 84.3 [83.1-85.4] | 5,961 | 89.6 (88.9-90.3) | 89.8 [89.0-90.5] |
| Independent | 974 | 9.2 (8.7-9.8) | 9.8 [9.2-10.4] | 523 | 13.4 (12.4-14.5) | 13.3 [12.2-14.4] | 451 | 6.8 (6.2-7.4) | 6.8 [6.2-7.4] |
| Not known (N/A) | 341 | 3.2 (2.9-3.6) | 3.0 [2.7-3.3] | 100 | 2.6 (2.1-3.1) | 2.4 [2.0-3.0] | 241 | 3.6 (3.2-4.1) | 3.5 [3.0-3.9] |
| School type |  |  |  |  |  |  |  |  |  |
| Primary school | 23 | 0.2 (0.15-0.3) | 0.3 [0.2-0.4] | 9 | 0.2 (0.1-0.4) | 0.3 [0.1-0.5] | 14 | 0.2 (0.1-0.4) | 0.3 [0.2-0.5] |
| Secondary school | 10,204 | 96.6 (96.3-97.0) | 96.8 [96.5-97.1] | 3,802 | 97.4 (96.8-97.8) | 97.5 [96.9-97.9] | 6,402 | 96.2 (95.7-96.7) | 96.3 [95.9-96.8] |
| Further education | 333 | 3.2 (2.8-3.5) | 2.9 [2.6-3.2] | 96 | 2.5 (2.0-3.0) | 2.3 [1.9-2.9] | 237 | 3.6 (3.1-4.0) | 3.4 [3.0-3.9] |
| School type - gender | | |  |  |  |  |  |  |  |
| % of mixed | 7,423 | 70.3 (69.4-71.2) | 70.8 [69.9-71.7] | 2,885 | 73.8 (72.4-75.2) | 73.9 [72.5-75.3] | 4,538 | 68.2 (67.1-69.3) | 68.1 [67.0-69.3] |
| School index of multiple deprivation - quintiles | | | |  |  |  |  |  |  |
| 1^st^ most deprived | 497 | 4.7 (4.3-5.1) | 4.8 [4.4-5.3] | 119 | 3.1 (2.6-3.7) | 3.4 [2.8-4.0] | 378 | 5.7 (5.1-6.2) | 6.1 [5.5-6.7] |
| 2^nd^ quintile | 1,905 | 18.0 (17.3-18.8) | 18.2 [17.5-19.0] | 567 | 14.5 (13.4-15.7) | 15.3 [14.2-16.6] | 1,338 | 20.1 (19.1-21.0) | 20.8 [19.8-21.8] |
| 3^rd^ quintile | 1,008 | 9.6 (9.0-10.1) | 9.5 [8.9-10.1] | 408 | 10.4 (9.5-11.4) | 10.1 [9.3-11.2] | 600 | 9.0 (8.3-9.7) | 8.9 [8.3-9.7] |
| 4^th^ quintile | 1,944 | 18.4 (17.7-19.2) | 18.4 [17.6-19.2] | 797 | 20.4 (19.2-21.7) | 20.1 [18.8-21.4] | 1,147 | 17.2 (16.4-18.2) | 17.1 [16.2-18.0] |
| 5^th^ least deprived | 4,865 | 46.1 (45.1-47.0) | 46.1 [45.1-47.1] | 1,916 | 49.0 (47.5-50.6) | 48.8 [47.2-50.4] | 2,949 | 44.3 (43.2-45.6) | 43.7 [42.5-45.0] |
| Not known | 341 | 3.2 (2.9-3.6) | 3.0 [2.7-3.3] | 100 | 2.6 (2.1-3.1) | 2.4 [2.0-3.0] | 241 | 3.6 (3.2-4.1) | 3.5 [3.0-3.9] |

^a^Weighted to account differences in the distribution of selected sociodemographic variables between the study sample and the target population
^b^’Yes’ includes those who reported having experienced food poverty from ‘once or twice’ to ‘every day’
^c^Excludes 95 (0.9%) observations where data were missing

Clustering at the local authority, school or year group level did not inform calculation of the confidence intervals.

**Table 2: Prevalence of self-harm and care received, unadjusted and weighted proportions with 95% confidence intervals, by gender**

|  | **Total** | | | **Males** | | | **Females** | | |
| --- | --- | --- | --- | --- | --- | --- | --- | --- | --- |
|  | **N** | **Unweighted % (95% CI)** | **Weighted % [95% CI]^a^** | **N** | **Unweighted % (95% CI)** | **Weighted % [95% CI]^a^** | **N** | **Unweighted % (95% CI)** | **Weighted % [95% CI]^a^** |
| **Self-harm** |  |  |  |  |  |  |  |  |  |
| Lifetime | 1,457 | 13.8 (13.2-14.5) | 12.5 [11.9-13.1] | 285 | 7.3 (6.5-8.2) | 7.1 [6.3-8.0] | 1,172 | 17.6 (16.7-18.6) | 17.2 [16.3-18.1] |
| Past year | 1,133 | 10.7 (10.2-11.3) | 9.6 [9.1-10.2] | 206 | 5.3 (4.6-6.0) | 5.2 [4.5-5.9] | 927 | 13.9 (13.1-14.8) | 13.5 [12.7-14.4] |
| Past six months | 881 | 8.3 (7.8-8.9) | 7.4 [7.0-7.9] | 153 | 3.9 (3.4-4.6) | 3.8 [3.2-4.4] | 728 | 10.9 (10.2-11.7) | 10.6 [9.8-11.3] |
| During 1^st^ UK lockdown | 789 | 7.5 (7.0-8.0) | 6.7 [6.2-7.2] | 136 | 3.5 (3.0-4.1) | 3.4 [2.8-4.0] | 653 | 9.8 (9.1-10.6) | 9.5 [8.9-10.3] |
| **Ever received support for self-harm, % yes by source of support (of 1,457)^b^** | | | | |  |  |  |  |  |
| Parent, step-parent, or carer | 369 | 25.3 (23.2-27.6) | 25.0 [22.8-27.3] | 71 | 24.9 (20.2-30.3) | 24.2 [19.6-29.6] | 298 | 25.4 (23.0-28.0) | 25.2 [22.8-27.9] |
| Brother or sister | 112 | 7.7 (6.4-9.2) | 7.5 [6.3-9.1] | 24 | 8.4 (5.7-12.3) | 8.4 [5.6-12.3] | 88 | 7.5 (6.1-9.2) | 7.3 [5.9-8.9] |
| Someone else in your family | 54 | 3.7 (2.9-4.8) | 3.7 [2.8-4.8] | 11 | 3.9 (2.2-6.8) | 3.6 [2.0-6.4] | 43 | 3.7 (2.7-4.9) | 3.7 [2.7-5.0] |
| Friend(s) | 533 | 36.6 (34.1-39.1) | 35.9 [33.3-38.4] | 92 | 32.3 (27.1-37.9) | 31.4 [26.3-37.1] | 441 | 37.6 (34.9-40.4) | 37.5 [34.7-40.3] |
| GP (family doctor) | 115 | 8.0 (6.6-9.4) | 7.4 [6.2-8.9] | 18 | 6.3 (4.0-9.8) | 5.9 [3.7-9.1] | 97 | 8.3 (6.8-10.0) | 8.0 [6.6-9.7] |
| Social Worker | 64 | 4.4 (3.5-5.6) | 4.6 [3.6-5.9] | 15 | 5.3 (3.2-8.6) | 5.4 [3.2-8.8] | 49 | 4.2 (3.2-5.5) | 4.3 [3.2-5.7] |
| School or college nurse/welfare staff | 208 | 14.3 (12.6-16.2) | 13.6 [11.9-15.5] | 23 | 8.1 (5.4-11.9) | 8.0 [5.3-11.8] | 185 | 15.8 (13.8-18.0) | 15.6 [13.6-17.8] |
| Psychologist or psychiatrist | 152 | 10.4 (8.8-9.1) | 10.2 [8.7-11.9] | 26 | 9.1 (6.3-13.1) | 9.2 [6.3-13.3] | 126 | 10.8 (9.1-12.7) | 10.5 [8.9-12.4] |
| Telephone helpline | 63 | 4.3 (3.4-5.5) | 4.0 [3.1-5.1] | 6 | 2.1 (1.0-4.6) | 2.0 [0.9-4.3] | 57 | 4.9 (3.8-6.3) | 4.7 [3.6-6.1] |
| Drop-in/advice centre | 9 | 6.1 (3.2-11.8) | 6.3 [3.2-12.6] | 2 | 0.7 (0.2-2.8) | 0.8 [0.2-3.4] | 7 | 0.6 (0.3-1.3) | 0.6 [0.3-11.8] |
| Residential Warden | 3 | 0.2 (0.07-0.6) | 0.2 [0.07-0.6] | 1 | 0.4 (0.05-2.5) | 0.3 [0.05-0.2] | 2 | 0.2 (0.04-0.7) | 0.2 [0.04-0.6] |
| CAMHS | 184 | 12.6 (11.0-14.4) | 12.1 [10.5-13.9] | 27 | 9.5 (6.6-13.5) | 9.6 [6.6-13.8] | 157 | 13.4 (11.6-15.5) | 12.9 [11.1-15.0] |
| Website or online forum | 128 | 8.8 (7.4-10.4) | 8.6 [7.2-10.2] | 14 | 4.9 (2.9-8.1) | 5.3 [3.1-8.8] | 114 | 9.7 (8.2-11.6) | 9.8 [8.2-11.7] |
| No-one **^c^** | 548 | 37.6 (35.2-40.1) | 38.3 [35.7-40.9] | 121 | 42.5 (36.8-48.3) | 43.8 [38.1-49.8] | 427 | 36.4 (33.7-39.2) | 38.8 [36.0-41.7] |
| **How helpful has support received (of 1,457)** | | |  |  |  |  |  |  |  |
| Not helpful at all | 305 | 20.9 (18.9-23.2) | 20.9 [18.9-23.2] | 60 | 21.5 (16.7-26.2) | 21.7 [17.2-27.1] | 245 | 20.9 (18.7-23.3) | 20.7 [18.4-23.1] |
| Not helpful enough | 276 | 18.5 (17.0-21.0) | 18.5 [16.6-20.7] | 33 | 11.6 (8.3-15.6) | 11.3 [8.1-15.5] | 243 | 20.7 (18.5-23.2) | 21.1 [18.8-23.6] |
| Just about helpful | 329 | 22.6 (20.5-24.8) | 22.8 [20.7-25.2] | 74 | 26.0 (21.2-31.4) | 25.6 [20.8-31.0] | 255 | 21.8 (19.5-24.2) | 21.9 [19.5-24.4] |
| Quite helpful | 266 | 18.3 (16.4-20.3) | 18.0 [16.0-20.0] | 46 | 16.0 (12.3-20.9) | 15.9 [12.1-20.7] | 220 | 18.8 (16.6-21.1) | 18.7 [16.5-21.1] |
| Very helpful | 158 | 10.8 (9.4-12.6) | 11.3 [9.7-13.2] | 46 | 16.0 (12.3-20.9) | 16.3 [12.3-21.2] | 112 | 9.6 (8.0-11.4) | 9.6 [8.0-11.5] |
| Not known | 123 | 8.4 (7.1-10.0) | 8.4 [7.0-10.0] | 26 | 9.1 (6.3-13.1) | 9.2 [6.3-13.3] | 97 | 8.3 (6.8-10.0) | 8.1 [6.6-9.8] |
| **Why did not *receive* support? % yes (of 548 who did not receive support)^c^** | | | | | |  |  |  |  |
| 1.Did not trust anyone | 226 | 41.2 (37.2-45.4) | 40.0 [35.9-44.3] | 35 | 28.9 (21.5-37.7) | 27.7 [20.5-36.4] | 191 | 44.7 (40.1-49.5) | 45.2 [40.4-50.1] |
| 2.Did not want help | 320 | 58.4 (54.2-62.5) | 59.3 [55.0-63.5] | 82 | 67.8 (58.9-75.5) | 66.9 [57.8-74.9] | 238 | 55.7 (51.0-60.4) | 56.1 [51.3-61.8] |
| 3.Didn't want to burden anyone else | 303 | 55.3 (51.1-59.4) | 54.5 [50.2-58.8] | 59 | 48.8 (39.9-57.7) | 48.2 [39.2-57.2] | 244 | 57.1 (52.4-61.8) | 57.2 [52.4-61.9] |
| 4.Didn't want the stigma | 143 | 26.1 (22.6-30.0) | 25.0 [21.5-28.9] | 26 | 21.5 (15.0-29.8) | 20.5 [14.2-28.6] | 117 | 27.4 (23.4-31.8) | 27.0 [22.9-31.4] |
| 5.Didn't know where to get help | 67 | 12.2 (9.7-15.3) | 11.5 [9.1-14.4] | 7 | 5.7 (2.8-11.7) | 5.3 [2.5-10.7] | 60 | 14.1 (11.1-17.8) | 14.1 [11.1-17.8] |
| 6.Worried about it not being kept confidential | 232 | 42.3 (38.3-46.5) | 41.4 [37.2-45.7] | 41 | 33.9 (26.0-42.8) | 32.9 [25.0-41.8] | 191 | 44.7 (40.1-49.5) | 45.0 [40.3-49.9] |
| 7.Scared/worried about what people might say | 270 | 49.3 (45.1-53.5) | 48.0 [43.6-52.3] | 49 | 40.5 (32.1-49.5) | 39.7 [31.3-48.9] | 221 | 51.8 (47.0-56.5) | 51.4 [46.6-56.2] |
| 8.Other | 106 | 19.3 (16.2-22.9) | 19.3 [16.1-23.0] | 20 | 16.5 (10.9-24.3) | 17.2 [11.3-25.4] | 86 | 20.2 (16.6-24.2) | 20.5 [16.9-24.5] |
| **Needed treatment? % yes (of 1,457)^b^** | | |  |  |  |  |  |  |  |
| My own first aid | 779 | 53.5 (50.9-56.0) | 52.5 [49.8-55.1] | 113 | 39.7 (34.1-45.5) | 39.8 [34.2-45.7] | 666 | 56.8 (54.0-59.6) | 57.0 [54.1-59.8] |
| Family-provided first aid | 88 | 6.0 (4.9-7.4) | 5.6 [4.6-6.9] | 8 | 2.8 (1.4-5.5) | 2.6 [1.3-5.3] | 80 | 6.8 (5.5-8.4) | 6.7 [5.4-8.3] |
| School nurse/first aid at school/college | 59 | 4.0 (3.2-5.2) | 3.9 [3.0-5.0] | 7 | 2.5 (1.2-5.1) | 2.3 [1.1-4.7] | 52 | 4.4 (3.4-5.8) | 4.5 [3.4-5.9] |
| Friends helped me | 126 | 8.7 (7.3-10.2) | 8.5 [7.2-10.1] | 19 | 6.7 (4.3-10.2) | 6.2 [4.0-9.5] | 107 | 9.1 (7.6-10.9) | 9.4 [7.8-11.3] |
| GP (family doctor) | 42 | 2.9 (2.1-3.9) | 2.7 [2.0-3.7] | 6 | 2.1 (1.0-4.6) | 2.0 [0.1-4.2] | 36 | 3.1 (2.2-4.2) | 3.0 [2.2-4.1] |
| Ambulance/paramedics | 25 | 1.7 (1.2-2.5) | 1.6 [1.1-2.3] | 2 | 0.7 (0.2-2.8) | 0.8 [0.2-0.9] | 23 | 2.0 (1.3-2.9) | 1.8 [1.2-2.8] |
| Hospital A&E / acute mental health provision | 52 | 3.6 (2.7-4.7) | 3.4 [2.6-4.5] | 12 | 4.2 (2.4-7.3) | 3.9 [2.2-6.8] | 40 | 3.4 (2.5-4.6) | 3.3 [2.4-4.4] |
| Hospital with overnight stay on ward | 43 | 3.0 (2.2-4.0) | 2.8 [2.1-3.8] | 8 | 2.8 (1.4-5.5) | 3.0 [1.5-5.9] | 35 | 3.0 (2.2-4.1) | 2.8 [2.0-3.9] |
| Other | 126 | 8.7 (7.3-10.2) | 8.5 [7.2-10.1] | 25 | 8.8 (6.0-12.7) | 8.7 [5.9-12.6] | 101 | 8.6 (7.1-10.4) | 8.4 [7.0-10.2] |

^a^Weighted to account for differences in the distribution of selected sociodemographic variables between the study sample and the target population
^b^Can include more than one response
^c^Includes only those who sought no support

Clustering at the local authority, school or year group level did not inform calculation of the confidence intervals.

**Figure 2: Source of support accessed following self-harm , weighted proportions^a^**

**Figure 3: Level of satisfaction with support received by source of support, weighted proportions^a^**

^a^Weighted to account for differences in the distribution of selected sociodemographic variables between the study sample and the target population.

**Supplementary Table 1: Self-harm questionnaire**

|  | Item | Response options |
| --- | --- | --- |
| 1 | Have you ever deliberately self-harmed (for example by taking an overdose or deliberately injuring yourself in some other way)? | Yes/ No/ Not sure what this means |
| 2 | Have you ever deliberately injured yourself in some way? | Never/ Once or twice/ A few times/ Weekly/ Daily |
| 3 | Has this happened during lockdown? | Never/ Once or twice/ Monthly/ Weekly/ Most days |
| 4 | When did you last self-harm? | In the last week/ In the last month/ In the past 3-6 months/ 6 months to a year ago/ Over a year ago |
| 5 | Have you ever deliberately taken an overdose (e.g., of pills or other medication)? | Never/ Yes – Once/ Yes – On more than one occasion |
| 6 | Has this happened during lockdown? | Never/ Yes – Once/ Yes – On more than one occasion |
| 7 | When did you last take an overdose? | In the last week/ In the last month/ In the past 3-6 months/ 6 months to a year ago/ Over a year ago |
| 8 | The last time this happened, in what way did you self-harm/ overdose? | [Free text response with 100-character limit] |

**Supplementary Table 2: Characteristics of adolescents who self-harmed and received no support following their self-harm, unweighted and weighted proportions with 95% confidence intervals.**

|  | No support | | | Support | | |
| --- | --- | --- | --- | --- | --- | --- |
|  | N | Unweighted % (95% CI) | Weighted % [95% CI]^a^ | N | Unweighted % (95% CI) | Weighted % [95% CI]^a^ |
|  | 548 | 37.6 (35.2-40.1) | 38.3 [35.7-40.9] | 909 | 62.4 (59.9-64.8) | 61.7 [59.1-64.3] |
| Sociodemographic characteristics | |  |  |  |  |  |
| Males | 121 | 42.5 (36.8-48.3) | 43.2 [37.4-48.3) | 164 | 57.5 (51.7-63.2) | 56.8 [50.8-62.6] |
| Females | 427 | 36.4 (33.7-39.2) | 36.5 [33.8-39.4] | 745 | 63.6 (60.8-66.3) | 63.5 [60.6-66.2] |
| School year |  |  |  |  |  |  |
| Year 8-9 (age 12-14 years) | 221 | 38.0 (34.2-42.1) | 38.3 [34.3-42.5] | 360 | 62.0 (57.9-65.8) | 61.7 [57.5-65.7] |
| Year 10-11 (age 14-16 years) | 208 | 38.0 (34.0-42.1) | 38.9 [34.7-43.2] | 340 | 62.0 (57.9-66.0) | 61.1 [56.8-65.3] |
| Year 12-13 (age 16-18 years) | 119 | 36.3 (31.2-41.6) | 37.3 [32.0-42.9] | 209 | 63.7 (58.3-68.8) | 62.7 [57.1-68.0] |
| Student born in the UK |  |  |  |  |  |  |
| Non-UK | 76 | 45.2 (37.9-52.9) | 45.8 [38.3-53.6] | 92 | 54.8 (47.2-62.2) | 54.2 [46.4-61.7] |
| UK | 471 | 36.7 (34.1-39.6) | 36.9 [34.2-39.6] | 813 | 63.3 (60.6-65.9) | 63.1 [60.4-65.8] |
| Unknown | 1 | 20.0 (2.1-74.4) | 26.8 [3.9-76.7] | 4 | 80.0 (25.6-97.9) | 73.3 [23.4-96.1] |
| Parents born in the UK |  |  |  |  |  |  |
| Non-UK | 217 | 44.1 (39.8-48.5) | 44.9 [40.3-49.5] | 275 | 55.9 (51.5-60.2) | 55.1 [50.5-59.7] |
| UK | 325 | 34.3 (31.3-37.4) | 34.3 [31.3-37.4] | 622 | 65.7 (62.6-68.4) | 65.7 [62.6-68.7] |
| Unknown | 6 | 33.3 (15.4-57.8) | 34.2 [16.0-58.5] | 12 | 66.7 (42.2-84.6) | 65.8 [61.5-84.0] |
| Free school meals |  |  |  |  |  |  |
| No | 418 | 38.3 (35.4-41.2) | 39.2 [36.2-42.2] | 674 | 61.7 (58.8-64.6) | 60.9 [57.8-63.8] |
| Yes | 37 | 29.1 (21.9-37.7) | 28.2 [21.0-36.8] | 90 | 70.9 (62.3-78.1) | 71.8 [63.2-79.0] |
| Not known | 93 | 39.1 (33.1-45.4) | 39.7 [33.4-46.3] | 145 | 60.9 (54.6-66.9) | 60.3 [53.7-66.6] |
| Ever experienced food poverty |  |  |  |  |  |  |
| No | 432 | 37.3 (34.5-40.1) | 37.9 [35.1-40.9] | 727 | 62.7 (59.9-65.5) | 62.1 [59.1-64.9] |
| Yes^b^ | 94 | 38.4 (32.5-44.6) | 39.0 [32.8-45.5] | 151 | 61.6 (55.4-67.5) | 61.0 [54.5-67.2] |
| Not known | 22 | 41.5 (29.0-55.2) | 42.9 [30.0-56.8] | 31 | 58.5 (44.8-71.0) | 57.1 [43.2-70.0] |
| Mental health |  |  |  |  |  |  |
| Symptoms of depression (RCAD_D), mean (95% CI)^c^ (n=1,454) | 547 | 65.3 (64.2-66.4) | 64.8 [63.6-65.9] | 907 | 65.1 (64.2-65.9) | 64.9 [64.1-65.8] |
| Symptoms of anxiety (RCAD_A), mean (95% CI)^c^ (n=1,454) |  | 60.5 (59.5-61.6) | 60.3 [59.2-61.4] |  | 60.5 (59.5-61.6) | 60.9 [60.9-61.8] |
| Ever received mental health support | | |  |  |  |  |
| No | 343 | 52.9 (49.0-56.7) | 53.2 [49.2-57.1] | 306 | 47.2 (43.3-51.0) | 46.9 [42.8-50.9] |
| Yes | 204 | 25.5 (22.5-28.5) | 25.5 [22.6-28.8] | 600 | 74.6 (71.5-77.5) | 74.5 [71.2-77.4] |
| Not known | 1 | 25.0 (2.4-82.0) | 22.4 [2.9-73.8] | 3 | 75.0 (18.0-97.6) | 77.6 [26.2-97.1] |
| School characteristics |  |  |  |  |  |  |
| Rural/urban |  |  |  |  |  |  |
| Rural | 84 | 32.2 (26.8-38.1) | 32.2 [26.7-38.2] | 177 | 67.8 (61.9-73.2) | 67.9 [61.8-73.4] |
| Urban | 464 | 38.8 (36.0-41.6) | 39.6 [36.7-42.5] | 732 | 61.2 (58.4-63.9) | 60.5 [57.5-63.3] |
| Funding source |  |  |  |  |  |  |
| State funded | 481 | 36.9 (34.3-39.5) | 37.6 [34.9-40.3] | 823 | 63.1 (60.5-65.7) | 62.4 [59.7-65.1] |
| Independent | 43 | 46.7 (36.8-57.0) | 47.2 [36.8-57.8] | 49 | 53.3 (43.0-63.3) | 52.8 [42.2-63.2] |
| Not known (N/A) | 24 | 39.3 (27.9-52.1) | 39.2 [27.7-52.0] | 37 | 60.7 (47.9-72.1) | 60.8 [48.0-72.3] |
| School type |  |  |  |  |  |  |
| Primary school | 1 | 25.0 (2.4-82.0) | 16.2 [2.0-65.1] | 3 | 75.0 (18.0-97.6) | 83.8 [34.9-98.0] |
| Secondary school | 523 | 37.5 (35.0-40.1) | 38.3 [35.7-41.0] | 871 | 62.5 (59.9-65.0) | 61.7 [59.0-64.3] |
| Further education | 24 | 40.7 (28.9-53.7) | 40.5 [28.7-53.5] | 35 | 59.3 (46.3-71.1) | 59.5 [46.5-71.3] |
| School type - gender |  |  |  |  |  |  |
| Boys | 33 | 47.8 (36.3-59.6) | 49.9 [38.0-61.7] | 36 | 52.2 (40.4-63.7) | 50.2 [38.3-62.0] |
| Girls | 126 | 41.2 (35.8-46.8) | 41.3 [35.8-46.9] | 180 | 58.8 (53.2-64.2) | 58.8 [53.1-64.2] |
| Mixed | 389 | 36.0 (33.1-38.9) | 36.6 [33.6-39.6] | 693 | 64.1 (61.1-66.9) | 63.5 [60.4-66.4] |
| School index of multiple deprivation - quintiles | | | |  |  |  |
| 1^st^ most deprived | 21 | 32.8 (22.4-45.3) | 31.1 [21.0-43.4] | 43 | 67.2 (54.7-77.6) | 68.9 [56.6-79.0] |
| 2^nd^ quintile | 115 | 52.4 (36.7-48.4) | 42.7 [36.8-49.0] | 156 | 57.6 (51.6-63.3) | 57.3 [51.0-63.2] |
| 3^rd^ quintile | 58 | 38.4 (31.0-46.4) | 39.0 [31.4-47.3] | 93 | 61.6 (53.6-69.0) | 61.0 [52.7-68.7] |
| 4^th^ quintile | 108 | 39.0 (33.4-44.9) | 39.8 [34.0-45.9] | 169 | 61.0 (55.1-66.6) | 60.2 [54.1-66.0] |
| 5^th^ least deprived | 222 | 35.1 (31.4-38.9) | 36.1 [32.3-40.0] | 411 | 64.9 (61.1-68.6) | 63.9 [60.0-67.8] |
| Not known | 24 | 39.3 (27.9-52.1) | 39.2 [27.7-52.0] | 37 | 60.7 (47.9-72.1) | 60.8 [48.0-72.3] |

^a^Weighted to account differences in the distribution of selected sociodemographic variables between the study sample and the target population
^b^’Yes’ includes those who reported having experienced food poverty from ‘once or twice’ to ‘every day’
^c^Excludes 95 (0.9%) observations where data were missing

Clustering at the local authority, school or year group level did not inform calculation of the confidence intervals.
